# Life history impacts on infancy and the evolution of human social cognition

**DOI:** 10.3389/fpsyg.2023.1197378

**Published:** 2023-11-09

**Authors:** Kristen Hawkes

**Affiliations:** Department of Anthropology, College of Social and Behavioral Science, University of Utah, Salt Lake City, UT, United States

**Keywords:** hominid comparisons, maturation rates, birth intervals, feeding dependence, sibling rivalry, parent-offspring conflict, neural development, brain size

## Abstract

Greater longevity, slower maturation and shorter birth intervals are life history features that distinguish humans from the other living members of our hominid family, the great apes. Theory and evidence synthesized here suggest the evolution of those features can explain both our bigger brains and our cooperative sociality. I rely on Sarah Hrdy’s hypothesis that survival challenges for ancestral infants propelled the evolution of distinctly human socioemotional appetites and Barbara Finlay and colleagues’ findings that mammalian brain size is determined by developmental duration. Similar responsiveness to varying developmental contexts in chimpanzee and human one-year-olds suggests similar infant responsiveness in our nearest common ancestor. Those ancestral infants likely began to acquire solid food while still nursing and fed themselves at weaning as chimpanzees and other great apes do now. When human ancestors colonized habitats lacking foods that infants could handle, dependents’ survival became contingent on subsidies. Competition to engage subsidizers selected for capacities and tendencies to enlist and maintain social connections during the early wiring of expanding infant brains with lifelong consequences that Hrdy labeled “emotionally modern” social cognition.

## Introduction

My evolutionary perspective assumes that features of living things are the result of a history of natural selection in the past (Fisher, 1930; Williams, 1966a; Grafen, 1988; Hawkes, 2006a; O'Connell and and Hawkes, 2023). Phylogeny always matters because selection can only favor what is present at the time. That explains why we share features with other members of the mammalian radiation and more with members of our primate order. We share even more with members of our hominid family, chimpanzees and the other great apes. Comparisons between us and them identify changes that evolved as our lineages diverged from the most recent ancestor we share. Compared to all living great apes, humans have bigger brains, longer lives, slower maturation, and shorter birth intervals. We also form lasting pair-bonds and our children require provisioning. The favored explanation for the evolution of these differences has long been the hunting/paternal provisioning/nuclear families as units of common interest hypothesis which I summarize below followed by some counterevidence before reviewing an alternative hypothesis about the evolution of human life history that is privileged here. The label “life history” is used differently in different contexts. I am using the demographic definition of evolutionary/ behavioral ecology: age specific fertility and age specific survival across the lifespan (e.g., Partridge and Harvey, 1988) and the body of theory and empirical findings about the wide variation in those population vital rates across the living world. After summarizing a grandmother hypothesis which draws from that theory and body of empirical comparisons to explain the evolution of human life history, I focus on novel survival challenges for ancestral youngsters entailed by their dependence on food they could not acquire for themselves. Subsidies from others meant their mothers had higher reproductive success with shorter birth intervals, shorter intervals which increased competition among dependents for attention and support. Hrdy (1999) identified those challenges to ancestral youngsters almost a quarter of a century ago and used them to explain the evolution of precocious sociality in human infants who otherwise develop so slowly (Hrdy, 2009). I combine Hrdy’s insights with comparative evidence collected by Barbara Finlay and colleagues that variation in brain size and composition across the whole radiation of placental mammals is explained by variation in the duration of development (Finlay and Darlington, 1995; Finlay et al., 2010; Charvet and Finlay, 2012; Workman et al., 2013). Longer developmental duration expanded human brains and intersected with shorter birth intervals (Finlay and Uchiyama, 2015). The intersection increased novel survival challenges for ancestral infants because unlike the infants ancestral to them, their survival depended not just on mothers’ milk but also on additional subsidies. That dependence put infants under strong selection for capacities and tendencies to engage and maintain subsidizing relationships while their slower developing brains were notably immature. Survival benefits for prioritizing relationships wired precocious sociality into ancestral human infants with consequences for our lifelong sensitivity to cultural contexts (Hawkes, 2020a).

The long-favored explanation for the evolution of our human genus is resisted here. That hunting/ paternal provisioning/ nuclear families as units of common interest hypothesis proposes that spreading savannas in ancient Africa were an opportunity for some ancestral hominins to begin hunting the big herbivores thriving in those savannas. Since babies interfere with hunting, ancestral females did better to pair with males who hunted to provision them and their offspring (Washburn and Lancaster, 1968; Lancaster and Lancaster, 1983). It was paternal provisioning that allowed ancestral mothers to bear another baby well before the previous one could feed itself (Lovejoy, 1981); and reliance on paternal provisioning favored the evolution of bigger brains and longer juvenile dependency to learn and practice skills that increased hunting success, supporting more offspring (e.g., Kaplan et al., 2000; Kaplan and Robson, 2002; Richerson and Boyd, 2020). As summarized in the classic text (Washburn and Lancaster, 1968, p. 301):

“… the habitual sharing between a male, a female, and their offspring becomes the basis for the human family. According to this view, the human family is the result of the reciprocity of hunting, the addition of a male to the mother-plus-young social group of the monkeys and apes….

To see how radically hunting changed the economic situation, it is necessary to remember that in monkeys and apes an individual simply eats what it needs. After an infant is weaned, it is on its own economically and is not dependent on adults.”

Since the field defining volume, *Man the Hunter* (Lee and deVore, 1968) in which that summary appeared, much more has been learned about the ways of living people who depend on foraging for subsistence and about the ways of our great ape cousins, both in the wild and in captivity. Although Paleolithic archeology was initially seen to support the hunting hypothesis, archeologists have since raised substantial challenges (e.g., Binford, 1981; O'Connell et al., 1988, 2002, review in Hawkes, 2016). Challenges from ethnographer James Woodburn (1968) had already been aired in one of his chapters in the *Man the Hunter* volume itself, which summarized his findings about Hadza hunters in Northern Tanzania where the big ungulates and carnivore competitors provide a living analog for aspects of the ecology in which our genus evolved. Woodburn reported that Hadza nuclear families are *not* units of common economic interest. Men rarely succeed at acquiring large game. Plant foods provide the bulk of Hadza diets, which men gather for themselves while hunting big game; women forage separately to collect reliable plant foods that feed them and their children. Ethnographies of other living people depending on wild foods, solving daily subsistence problems much more ancient than the origins of agriculture find that men tend to prioritize activities that contribute to their social standing relative to other men (e.g., Hawkes, 1991, 1992, 1993, 2000). Yet the long-recognized pattern of persistent human pair-bonding across subsistence systems (e.g., Murdock, 1949, 1967; Muller and Pilbeam, 2017) along with the earliest archeology indicating ancestors’ butchery of big game carcasses seemed to provide overwhelming support for the hunting hypothesis, including the influential version that Marshall Sahlins labeled “The Domestic Mode of Production” (Sahlins, 1972). Like the ubiquity of persistent pair bonds, the importance of schools in *our* socioecology tends to favor continuing assumptions that teaching, skills-learning and long practice have been central in the evolution of human life history. Yet children on their own quickly pick up behaviors to fit in with peers (e.g., Harris, 1995; Harris, 1998; Flinn and Ward, 2005). Careful quantitative ethnographies have found that among hunter-gatherers differences between adult and juvenile foraging are better explained by size and strength than by needs for teaching or long practice (e.g., Bird and Bird, 2000; Bird and Bliege Bird, 2002; Bliege Bird and Bird, 2002; Blurton Jones and Marlowe, 2002).

## A grandmother hypothesis

An alternative hypothesis similarly begins with the ecological changes that occurred in ancient Africa. But it highlights the spread of plants that coped with stronger seasonal swings in rainfall. For plants that endure dry seasons by sequestering water and starch in underground storage organs (USOs) are foraging opportunities for consumers that can access them. However, access from the surface for many of these resources requires size and strength. In contrast to the easier pickings of the soft fruits and leaves of the forest that great apes rely on, small youngsters cannot extract the USOs that give high reliable return rates to adults. Modern Hadza foragers illustrate both the problem and a solution, lessons directly relevant to the evolution of human life history (Hawkes et al., 1989, 1995, 1997, 2001, 2018). In our small initial sample of observations Hadza mothers’ foraging usually supported their dependent children. But when mothers had a newborn, support for weaned dependents came from grandmothers. If ancestral grandmothers’ foraging productivity was a reliable subsidy for mothers’ weaned dependents, then selection would favor ancestral mothers giving birth to the next infant sooner. Ancestral grandmothers that were aging slightly more slowly could provide more support, selection then favoring slower aging, longer lifespans and still shorter birth intervals in subsequent generations. Observations of that “division of child rearing labor” among modern Hadza women prompted our version of a “grandmother hypothesis” to explain the evolution of distinctive human life histories (Hawkes et al., 1997, 1998; O'Connell et al., 1999; Hawkes, 2003, 2010; Hawkes and Coxworth, 2013). Blurton Jones’s (2016, p. 359–382) much larger demographic data set and analyses are consistent with this hypothesis. Here I highlight the novel selection on socioemotional cognition beginning in infancy that is entailed when two things are explicitly incorporated into that grandmothering scenario.^1^ Those two things are Sarah Hrdy’s insights about the likely role of sibling rivalry when longer offspring dependence on support combines with shorter birth intervals, and Barbara Finlay and colleagues’ findings that it is slower duration of development that expands brain size across the placental mammals including humans. First comes a bit more theoretical and comparative background.

The groundwork for an increased postmenopausal longevity hypothesis had been laid earlier in Eric Charnov’s modeling of life history evolution across the mammals (Charnov, 1990, 1993; Charnov and Berrigan, 1993). In Charnov’s model, adult mortality (the inverse of longevity) accounts for the mouse to elephant range of variation in the length of time juveniles continue to grow bigger before reaching maturity. Lower adult mortality favors delaying maturity as larger mothers can put more into offspring. But the cost of waiting is the risk of dying first. When that risk goes down, selection favors longer delay, slowing development, and slowing the rate of baby production as more goes into each. Comparisons between humans and chimpanzees (and other great apes) are only consistent with that framework IF our greater longevity, slower maturation and shorter birth intervals evolved as women’s post-fertile life stage subsidized the fertility of childbearing years. Duration of development *is* shorter in non-human hominids; they grow old faster, females usually dying while still fertile (e.g., Goodall, 1986; Emery Thompson et al., 2007). Human development *is* slower, and women who survive to adulthood *usually* out-live their fertility (Howell, 1979; Blurton Jones et al., 1992; Hill and Hurtado, 1996; Hawkes, 2003; Gurven and Kaplan, 2007; Blurton Jones, 2016). Consistent with that grandmother hypothesis, human rates of baby production *are* much higher than Charnov’s model predicts for a non-grandmothering mammal with our longevity (Hawkes et al., 1998; O'Connell et al., 1999; Robson et al., 2006; Hawkes, 2006b). If ancestral females *were* subsidizing the fertility of their daughters as their own fertility was ending, slower aging would raise the reproductive rate of their descendants (Hamilton, 1966).

Peter Kim’s two-sex agent-based model of that grandmother hypothesis simulated the evolutionary process. An ancestral chimpanzee-like age structure evolved to be human (hunter-gatherer)-like with about a third of the adult females past their fertility (Kim et al., 2012, 2014, 2019). The formal modeling also provided an initially unrecognized explanation for the persistent pair-bonding that distinguishes humans from the other great apes (and most mammals). As longevity increases, the older age structure of the population includes not only postmenopausal women but also still-fertile old men. With more competitors, the strategy that wins more paternities shifts from competing for sequential conception possibilities with different partners, mating multiply, to claiming a mate and persistently guarding her (Hawkes and Coxworth, 2013; Coxworth et al., 2015; Schacht and Bell, 2016; Loo et al., 2017a,b, 2020, 2021; Rose et al., 2019). Pervasiveness of proprietary mate-guarding throughout ethnography and history has been widely recognized (e.g., Wilson and Daly, 1992, 1993; Smuts, 1995; Hrdy, 1997; Mesnick, 1997; Wilson and Mesnick, 1997; Hawkes, 2004), although it has regularly been explained as protection against misdirecting paternal effort (e.g., Daly et al., 1982). Yet that assumption of ubiquitous paternal support is undercut by facultative fathering (e.g., Hrdy, 2008) as well as male ontogeny and sexual dimorphism (e.g., Bribiescas, 2006; Puts, 2010, p. 161–163; and references therein) which indicate an evolutionary legacy of male mating competition that is directly inconsistent with a history of obligate paternal care. Instead, the male-biased sex ratio in the fertile ages that evolved with our grandmothering life history is a firmer foundation for the evolution of pair-bonding due to paternity advantages from persistent mate guarding (Coxworth et al., 2015; Loo et al., 2017a,b, 2020, 2021).

## Human longevity, big brains, and proper scaling

According to Charnov’s demographic life history modeling the fitness benefit of delayed maturity is continued growth to larger size as bigger moms put more into reproduction. Across the wide mammalian variation in adult size (mouse to elephant) both average adult lifespan and age at maturity increase with adult size at the same allometric rate. Charnov’s modeling also includes a ‘production coefficient’ that captures the variation in both growth rates and rates of offspring production and their correlation with each other across mammalian lineages. As Hawkes and Finlay (2018, p. 63) summarized, this parameter explains:

“…why, for a given adult size, primates have greater longevity, later maturity and slower rate of offspring production than do most non-primate mammals (Charnov and Berrigan, 1993)…. Charnov's mammal model highlighted the empirical pattern that primate bodies grow slowly compared to non-primate mammals but did not deal with brains (Charnov and Berrigan, 1993).”

Separating bodies from brains and proper scaling are also part of Barbara Finlay and colleagues’ documentation of the tight regularity in the sequence of neural maturational events that links duration of development to both final brain size and composition in placental mammals from mice to humans (Workman et al., 2013, still evident in apparent outliers, Finlay and Huang, 2020). Integrating those findings with demographic life history, Hawkes and Finlay (2018) said:

“For primates overall, the best account of the evolutionary progression of brain size and body size is that body size was first reduced, producing relatively large brain sizes …Since relatively larger brain size can arise from selection for smaller body size (Smaers et al., 2012), widely used measures of brain size relative to body size, like encephalization indices, that incorporate both effects can obscure the separate roles of each” (p 56).

“…studying variation across species of different sizes and developmental durations requires care. …the laws of space and time, of physics and chemistry, impose changes in both form and process as size and time change, making allometries a subject of long interest…. Considering only brain mass, the cortex has positive allometry with respect to the rest of the brain, a slope greater than one. Thus, larger mammalian brains become progressively more composed of cortex, ranging from under 20% in relative volume in small shrews and rodents to over 80% in humans” (p 57).

“In addition, …a further important issue in the accuracy of such comparisons is what point in development represents ‘zero,’… Although birth is often chosen as a natural zero, this choice can be very misleading. The range of maturational states at birth in mammals, including primates, is wide. *Time from conception, not birth*, proves to best explain variation in brain maturational state (Finlay and Darlington, 1995; Workman et al., 2013)” (p. 58) [emphasis added].

“…the mouse takes only about 30 days to execute its neuro developmental 271 events, while the human takes 1000 days for the same 271, humans generating greater numbers of neurons and volumes of connectivity per event. The fit of model results to empirically-measured results is astonishingly close, 0.9929…” (p. 59).

Summarizing these findings with respect to human infancies, Finlay and Uchiyama (2015, p. 129) drew two conclusions:

“First, …though it is entirely accurate to say that humans have the longest period of brain development of primates, as this parameter is virtually perfectly correlated with brain size (Workman et al., 2013), the claim that humans have been specially selected for a long developmental duration is unjustified…a large brain is a necessary by-product of selection for extended development.

Second, the timing of birth is quite uncorrelated with neural maturation…. Some rodents (mice and rats) are born at a stage of maturation equivalent to a human of 4–5 months gestation, whereas others like the guinea pig correspond to a human of approximately 3 years postnatal.”

That range of maturation at birth from altricial to precocial depends on maternal capacities and newborn challenges which vary independently of the demographic variables in Charnov’s modeling. Weaning age not gestation length is demographically crucial because that is when mothers can begin investing in the next baby (or litter). Also, in Charnov’s modeling proper scaling reveals the patterns. As Finlay and Uchiyama (2015) emphasized, earlier weaning age in humans compared to the other great apes has been commonly noted (e.g., recently Lonsdorf et al., 2020 for chimpanzees), but that is usually “based on absolute duration. If we examine allometric predictions for these species compared to brain maturation, we see that humans are weaned even earlier than the linear projection would suggest” (p. 142). This relatively early weaning combined with longer duration of neural development that is necessarily indicated by larger human brain size has huge consequences for selection on the socioemotional capacities and tendencies of ancestral infants.

“One of the most profound differences in human development … is the changed social environment produced by early weaning…. [T]he human child, unlike other primates, in the early parts of its “sensitive period” of development of any number of sensory, cognitive, motor, and social abilities [is thrust] out of the small society of mother and child … While much evidence suggests relatively greater attunement of the human child for social interaction, imitation, and cooperation…it is not only the motivations and preferences of the child that differ from its primate ancestors. Possessed of an exceptionally large brain … with an allometrically predictable extended period of maturation filtered by evolution to be permissive of variability, the human child exercises those motivations and preferences in social environments more variable in every respect than those of any immediate primate relative. The developmental niche we inhabit is thus a curious mixture of a conserved neurodevelopmental schedule and a specially adapted life history” (Finlay and Uchiyama, 2015, p. 143).

## Human cognitive specialties

That “curious mixture” is directly relevant to cognitive distinctions between us and chimpanzees that Michael Tomasello’s numerous experiments and syntheses have done so much to highlight (e.g., Tomasello et al., 2005; Hrdy, 2009, 2016; Tomasello, 2009, 2010, 2019, 2022; Hawkes, 2012, 2014). Tomasello and Carpenter (2007:121) explained the human distinction this way:

“…a suite of social-cognitive and social-motivational skills that may be collectively termed shared intentionality. Shared intentionality, sometimes called ‘we’ intentionality, refers to collaborative interactions in which participants share psychological states with one another…. For example, in problem-solving activities participants may have a shared goal and shared action plans for pursuing that goal, and in communication they may simply share experience with one another linguistically.”

The fitness benefit repeatedly identified for this capacity is the widespread cooperation it enables. To honor Lev Vygotsky’s (1978) focus on cultural transmission in child development, Tomasello labels this shared intentionality hypothesis “Neo-Vygotskyian,” and he and colleagues refer to it as “The cultural intelligence hypothesis” (Herrmann et al., 2007).

Cultural cooperation may have advantages for humans, but differences evident now between us and chimpanzees were not there when our radiations diverged. As Hrdy (2005, 2009) has noted, since natural selection is not forward looking, advantages evident in the present do not explain why more cooperative appetites evolved in us and not them. Tomasello subsequently agreed (Tomasello and Gonzalez-Cabrera, 2017), then considering that question: “why us, not them?” later, said:

“The compelling answer proposed by Hrdy (2009, 2016) and Hawkes (2014) [and elaborated by Tomasello and Gonzalez-Cabrera (2017)] is that at some point in evolution humans, but not other apes, switched to a system of cooperative childcare in which infants’ relationships to many non-mother adults became crucially important as well. Because this system also led to greater fecundity, the result was that human infants had to compete with a greater number of siblings and peers for the care of a greater number of adults, including at a distance. In this view, emotion sharing and other forms of shared intentionality emerge early in human ontogeny because evolutionarily, infants who possessed them were better able to bond with (and so receive more care and attention from) more adults, and at a distance…” (Tomasello 2019, p. 307).

That is a useful summary, but does it answer the “why us not them” question? Why cooperative childcare in us and not them? Before addressing that question, consider the contradictory foundation Tomasello assumed earlier in the same book:

“It is widely accepted among virtually all students of human evolution that the patterns of a relatively slow ontogeny, including slow brain growth, is at least partly an adaptation to humans’ cultural way of life, in which developing children have much to learn and many skills to develop before they can become competent members of their cultural group” (Tomasello 2019, p. 27).

A Vygotskyian presumption of human’s pre-existing “cultural way of life” cannot explain why the human distinctions began to evolve in the first place. The hypothesis I favor here is that distinctive “social-cognitive and social-motivational skills” evolved as a part of what Finlay and Uchiyama (2015, p. 143) called our “curious mixture of a conserved neurodevelopmental schedule and a specially adapted life history.”

Responsiveness to developmental context in children has long been of particular interest to anthropologists and some cross-cultural psychologists (e.g., Draper, 1976, 1978; Blurton Jones et al., 1979; Weisner, 1987; Hewlett, 1989, 1991, 1996; Blurton Jones et al., 1992, 2002; Blurton Jones, 1993; Harris, 1995; Harris, 1998; Keller, 2007, 2013; Rogoff, 2014; Lancy, 2016, 2018; Meehan and Crittenden, 2016; Keller and Bard, 2017). Emphasis has often been on variation in parenting styles; the important role of infant agency in setting cognitive priorities is emphasized here (e.g., Blurton Jones, 1972; Blurton Jones and da Costa, 1987; Haig, 2014). As Heidi Keller (2007) synthesizing much cross-cultural variation in parenting concluded, “infants are not the passive recipients of [parental lessons]…They process information actively and construct their psychology and their selves” (p. 256). Cross-cultural variation has been given less attention in experimental and developmental psychology where infant subjects have usually been those most conveniently available to research labs. Yet the error of tacit assumptions that infants are not yet affected by developmental context has long been recognized (e.g., Beach, 1950; Arnett, 2008; Henrich et al., 2010; Dahl, 2017; Nielsen et al., 2017). Variation in chimpanzee behavior has also been increasingly recognized with growing numbers of study sites (e.g., McGrew, 2017; Stanford, 2018; Hunt, 2020) and captive contexts (e.g., Bard, 2012; Matsuzawa, 2013).

Kim Bard et al. (2021) have now added to the evidence of variation with developmental context in both species by analyzing videos of one-year-old infants in three chimpanzee and three human groups differing in habitual infant social relationships. They coded 10-s intervals of 40-min videos for the occurrence of joint attention, identified this way (p. 10):

“Joint attention is a triadic ability in which infants coordinate attention to an object with attention to a social partner. Given the vast differences in human cognition compared to that of the great apes, joint attention is also thought to mark social cognition that is distinctly human (e.g., Tomasello, 2019).”

To be inclusive Bard and colleagues assay triadic coordination by documenting instances of joint engagement, defined as.

“…observable markers indicating that infants are engaged both with a partner and with an event or object. Although gaze alternation is often used to document that the infant is coordinating attention between the social partner and the object of shared attention, coordination in engagement can be obtained through other modalities Our definition allows for but does not require visual attention to mark the jointness of engagement” (p. 51, 52).

By identifying the variable frequency of joint engagement events among chimpanzee and human infants with varying developmental experience they further clarify this important aspect of infant social cognition. Although measurement is, as always, an issue, as is the notable variation among individuals within each group, the lesson of particular concern here is the overlapping variation they found between species. Based on that overlap, Bard et al. (2021, p. 190) “conclude that human-unique social cognitive development is not evident at 1 year of age.”

## Infant social capacities and life history evolution

More evidence of early similarity in potential infant social responsiveness in chimpanzees and humans adds force to the case that Sarah Hrdy made decades ago. In *Mother Nature*, Hrdy (1999) identified distinctive conflicts of interest between human mothers and offspring that result in our “ambivalent mothering” which contrasts with the more fully committed mothering of great apes. Human mothers, with overlapping dependents face novel tradeoffs over allocation of maternal investment. Other great ape mothers, supporting one infant until it transitions to entirely feeding itself can commit to each offspring sequentially. The human pattern of bearing another child while those born previously are still dependent on subsidies inflates what Robert Trivers (1974) called parent-offspring conflict, as he identified the inevitable “other side” of Hamilton’s rule (Hamilton, 1963, 1964).

WD Hamilton had used a simple inequality, rb − c > 0, to solve what he called a “riddle of altruism.” How could tendencies to supply benefits to others (b) at a cost to the benefactor (c) persist given that natural selection favors traits that increase an individual’s own reproductive success and so raise the frequency of an actor’s gene copies in future generations? Hamilton pointed out that just as parents benefitting offspring is parents benefitting copies of their genes, parent-offspring gene sharing is a special case of the more general reality of gene sharing among individuals within populations (r). The simple inequality of Hamilton’s rule identifies a threshold for what he called inclusive fitness, which selection is expected to maximize (Grafen, 1984). Trivers (1974) focused on the other side of the story. While inclusive fitness interests of kin overlap, the overlap is not perfect. In sexual reproducers offspring get half their genes from each parent. The chance that any (autosomal) gene in an offspring came from its mom is only 50%. Just as tendencies to give benefits that make more gene copies can be favored by selection, tendencies to take benefits can too. Hamilton’s rule is from a benefit giver’s perspective; from a taker’s perspective an inequality similar to it would be, b − rc > 0. Comparing both sides of a benefit transfer from mother to offspring (ignoring for simplicity the hugely important difference in the cs and bs) shows an offspring is selected to take four times the benefit that its mother is selected to give. Trivers laid out predictions for postnatal interactions including weaning conflict in mammals and sibling rivalry. David Haig (1993, 2019) carried the insight to the “battleground” of human pregnancy, especially the different calculus of costs to offspring and mother from the perspective of genes an offspring got from its father. In the other direction, siblings share more genes from their common parents than cousins share from common grandparents. Where individuals are gregarious, the usual pattern among primates, local groups include not only parents, offspring, and siblings but also cousins and others. When ancestral humans committed to habitats where infants and young juveniles cannot directly acquire the resources that all depend on for themselves, competition for the engagement of others became a matter of survival for those infants and youngsters. Sensitivities to calibrate and respond to social opportunities and dangers above all came under unprecedented selection. And, from the life history hypothesis privileged here, that was part of the slowed duration of development to larger brain size, that wired those sensitivities especially early in neural maturation.

Focusing here on mother-offspring and sibling relationships, each child gets half its genes from mom and siblings share only half of those. Nicholas Blurton Jones and Elizette da Cost (1987) suggested that sibling rivalry might explain behaviors of infant humans that likely delay mother’s next pregnancy [pursued again more recently by Haig (2014)]. Hrdy (1999) identified the inflation of sibling rivalry and selection for infant agency in response to inevitable “discriminative parental solicitude” (Daly and Wilson, 1980) with overlapping dependents. Discriminating maternal investment could account for the evolution of active flirtatiousness in helpless human babies. “Baby lust,” the intense interest in babies across our primate order (e.g., Hrdy, 1999, p. 61ff; Hrdy, 2009, p. 218ff and see Henzi and Barrett, 2002) was surely an aspect of the ancestral condition we share with other apes. It is the precocious sociality of human infants themselves, their active social agency *so at odds with the slowness of other aspects of development*, that convinced Hrdy “there must have been stringent selection among our ancestors for this infantile equivalent of sex appeal” (Hrdy, 1999, p. 484). She has subsequently elaborated the case that human reliance on allomothers underlies that stringent selection (e.g., Hrdy, 2005, 2007, 2009). Unlike the independent mothering of our very smart and very social chimpanzee cousins, some ancestral hominins began to rely on allomothers. Closer birth spacing then raised mothers’ fitness while at the same time increasing infant competition for support from mothers and others. Because subsidies were so critical for infant survival, that competition gave infants more successful at engaging mothers and others increased chances to survive and become our ancestors. Hrdy’s persuasive argument about selection on precocious sociality identifies ancestral dependence on allomothering as its foundation.

As she says, an explanation that points to *subsequent* benefits of uniquely human forms of cooperation begs the question:

“How did the emotional scaffolding facilitating mutual tolerance, interest in the mental states and thoughts of others, and eagerness to please and share with them emerge in the hominin line in the first place? It also fails to explain why these hyper-social impulses evolved in humans but not other apes” (Hrdy, 2016, p. 16).

A likely answer to that “why us?” question is the evolution of our grandmothering life history, the process simulated in Peter Kim’s modeling of the grandmother hypothesis (Kim et al., 2012, 2014, 2019). Hrdy (2014, 2016) suggested a thought experiment to clarify consequences that shortening birth intervals would have for selection on infant social agency. Consider the perspective of ancestral infants whose mothers begin bearing next babies while they are still entirely dependent. Their chance of survival rises if they can counter their mother’s reduced engagement and/or engage someone else. In an ecological context where youngsters are unable to feed themselves unlike the forests where *living great ape infants do that*, mother’s shift in attention to a newborn would have been an immediate survival challenge. I previously connected Hrdy’s insights about the likely effects of shorter birth spacing to the formal model of the grandmother hypothesis in Kim et al. (2012) this way:

“The model is, of course, an extreme simplification, aimed to see whether (very weak) helpful grandmothering could propel the evolution of a humanlike life history from an apelike one. It does not include the maternal problem of distributing attention among newborns and older dependent juveniles. … But there are never enough grandmothers in the model to take on all the juveniles eligible for nonmaternal help. If juveniles varied in their ability to engage that help, the mothers of those better able to do so would have shorter birth intervals and higher reproductive success” (Hawkes 2014, p. 32).

With ancestral infant survival on the line, selection on infant capacities and tendencies to attract support would be very strong (Hawkes, 2020a). Those likely consequences of shortening birth intervals underlined by Hrdy’s scenario are consistent with evidence that unusually short intervals to next birth impose costs on infants of other primates. They impact infant survival for rhesus macaques on Cayo Santiago (Lee et al., 2019) and for captive callitrichines (Frye et al., 2022). Short intervals are an “aversive childhood experience” impacting subsequent welfare as much as mother’s early death for Amboseli baboons (Zipple et al., 2019; see Morrison et al., 2021 for an alternative pattern in Mountain gorillas). Stress levels rise with the arrival of a sibling in chacma baboons in Namibia (Delaunay et al., 2023) and also in bonobos at LuiKotale (Behringer et al., 2022). In Kanyawara “chimpanzee mothers with the resources to do so prioritize production of new offspring over prolonged investment in current offspring,” even though “offspring growth suffers when mothers wean early to invest in new reproductive efforts” (Emery Thompson et al., 2016, p. 7,780).

For selection to favor shortened intervals *as the norm* implies a shift in ancestral mothers’ tradeoffs between current reproduction and somatic maintenance. Longer lived than other nonhuman primates, great apes are expected to allocate more to somatic maintenance. As multiyear lactators, nursing each infant across not only seasonal but annual resource variation, our great ape cousins set allocation to lactation relatively lower than shorter-lived primates do (van Noordwijk et al., 2013a,b). This is the expected consequence of a history of natural selection adjusting tradeoffs to maximize inclusive fitness (Hamilton, 1964). Benefits of somatic allocation depend on the likelihood of continued survival, i.e., adult mortality rates (Williams, 1957, 1966b; Kirkwood and Rose, 1991) and also on what survivors can do for their inclusive fitness (Williams, 1957; Hamilton, 1964, 1966; Hawkes, 2020b).

The grandmother hypothesis assumes that tradeoff: more to somatic allocation and less to current reproduction is reflected in the greater longevity and lower allocation to lactation in humans (Prentice and Prentice, 1988) and, even though youngsters cannot feed themselves (Sellen, 2006, 2014), shorter birth intervals, as tradeoffs during the childbearing years co-evolve with dependent subsidies during the postmenopausal ones (Kim et al., 2019). Intergenerational transfers shift selection on aging rates (Lee, 2003, 2008). Increased longevity slowed maturation with slower neural development expanding brain size (Finlay, 2019). That slower neural ontogeny intersected with earlier weaning (Hawkes and Finlay, 2018). As noted by Finlay and Uchiyama (2015, p. 143 cited above), social agency in human infants begins in the early parts of a “… ‘sensitive period’ of development of any number of sensory, cognitive, motor, and social abilities… it is not only the motivations and preferences of the child that differ from its primate ancestors….the human child exercises those motivations and preferences in social environments more variable in every respect than those of any immediate primate relative.”

## Concluding discussion

Behavior in both chimpanzees and humans varies with socioecological and developmental contexts. Characterizing that variation (e.g., Bard, 2012; Bard and Leavens, 2014; Keller and Bard, 2017; Keller, 2018; Bard et al., 2021) is important for improving hypotheses about selection on ancestral infants and the evolution of distinctly human socioemotional cognition (e.g., Hrdy, 2009; Tomasello, 2019; Hawkes, 2020a). Tomasello’s “shared intentionality” hypothesis and his ontogenetic inferences continue to meet skepticism from many primatologists. More observational attention paid to mother-offspring interactions in other apes (e.g., Reddy and Wellman, 2020; Tkaczynski et al., 2020; Heesen et al., 2021; Behringer et al., 2022) will continue to both complicate and clarify cross-species variation. As that work proceeds, the importance and explanatory reach of life history evolution is expanded by Sarah Hrdy’s insights about novel survival challenges faced by infants ancestral to us (Hrdy, 2005, 2009, 2014, 2016; Hawkes, 2014) especially when combined with the regularities in mammalian neural development identified by Finlay et al. (2010), Finlay and Workman (2013), Finlay and Uchiyama (2015), Hawkes and Finlay (2018), Finlay (2019), and Hawkes (2020a). Associations between weaned offspring and their mothers would surely have been important in ancestral populations as they are in chimpanzees now (e.g., Goodall, 1986; Nakamura et al., 2014; Crockford et al., 2020; Stanton et al., 2020), even though infants begin feeding themselves so early (e.g., Bădescu et al., 2017; Matsumoto, 2017; Bray et al., 2018). Ancestral commitment to habitats where not only infants but young juveniles could not feed themselves adequately must have escalated that importance. New siblings’ arrival would impose survival challenges that selected for social agency in infancy while expanding brains were still notably immature and physical capacities were developing more slowly.

As Hrdy (1999) explained, competition for maternal attention favored capacities and tendencies to be “an infant worth rearing” and, once kept, to “be adorable” and good at ingratiating oneself with others; or more generally doing what it takes to “fit in” (e.g., Lancy, 2016). Daily survival depending above all on “fitting in” socially was an evolutionary game changer. From the perspective of life history evolution, the game changed when members of ancestral populations took advantage of expanding foraging opportunities in spreading savannas. Competition with carnivores for big ungulate carcasses may have provided occasional bonanzas for all there (O'Connell et al., 2002; O'Connell and Hawkes, 2023), but those bonanzas could not have met daily consumption needs for anyone. However, plants like those that invested in USOs would be reliable resources year-round for foragers big and strong enough to extract them. Survival of small youngsters in that ecological context was contingent on reliable subsidies. According to the hypothesis favored here, that problem was solved by the foraging productivity of older females, which resulted in selection for extended postmenopausal longevity, slower maturation, and shortened birth intervals. That life history wired ancestral infants for active social agency early during the slower maturation of their expanding brains. Consequences for subsequent human experience are of great interest, but those consequences do not explain the initial evolution of our distinctive social appetites. Focus on the magnitude of subsequent benefits only begs the question: why us and not other great apes? The evolution of our grandmothering life history has comprehensive relevance to that question. From a demographic life history perspective enriched by consequences identified by Hrdy and by Finlay and colleagues, infancy comparisons should be especially valuable for improving hypotheses about the evolution of the distinctly cooperative social cognition that evolved in us and not the other great apes.

## Data availability statement

Publicly available datasets were analyzed in this study. All data referred to here have been previously published and primary citations are in the references.

## Ethics statement

The studies involving human participants were reviewed and approved by University of Utah Institutional Review Board. Written informed consent from the participants’ legal guardian/next of kin was not required to participate in this study in accordance with the national legislation and the institutional requirements.

## Footnotes

^1^Recognition that postmenopausal longevity increased in our genus contrasts with the “stopping early” hypothesis about the evolution of menopause that Williams (1957) suggested in his field defining paper on the evolution of senescence. Having explained why selection would not favor any “post reproductive” life, he then considered the apparent violation that postmenopausal women present to that expectation at a time when menopause was assumed uniquely human, no demographic data were available for modern hunter-gatherers, and little was known about either hominid phylogeny or the lives of our great ape cousins [review of that intellectual history in Hawkes (2020b)].
